# Development of an Orthotopic HPV16-Dependent Base of Tongue Tumor Model in MHC-Humanized Mice

**DOI:** 10.3390/pathogens12020188

**Published:** 2023-01-25

**Authors:** Christoph Schifflers, Samantha Zottnick, Jonas D. Förster, Sebastian Kruse, Ruwen Yang, Hendrik Wiethoff, Matthias Bozza, Karin Hoppe-Seyler, Mathias Heikenwälder, Richard P. Harbottle, Carine Michiels, Angelika B. Riemer

**Affiliations:** 1 Immunotherapy and Immunoprevention, German Cancer Research Center (DKFZ), 69120 Heidelberg, Germany; 2 Cell Biology Research Unit (URBC)–Namur Research Institute for Life Sciences (NARILIS), University of Namur, 5000 Namur, Belgium; 3 Molecular Vaccine Design, German Center for Infection Research, Partner Site Heidelberg, 69120 Heidelberg, Germany; 4 Faculty of Biosciences, Heidelberg University, 69120 Heidelberg, Germany; 5 Viral Transformation Mechanisms, German Cancer Research Center (DKFZ), 69120 Heidelberg, Germany; 6 Institute of Pathology, Heidelberg University Hospital, 69120 Heidelberg, Germany; 7 Helmholtz-University Group Cell Plasticity and Epigenetic Remodeling, German Cancer Research Center (DKFZ), 69120 Heidelberg, Germany; 8 DNA Vector Laboratory, German Cancer Research Center (DKFZ), 69120 Heidelberg, Germany; 9 Molecular Therapy of Virus-Associated Cancers, German Cancer Research Center (DKFZ), 69120 Heidelberg, Germany; 10 Chronic Inflammation and Cancer, German Cancer Research Center (DKFZ), 69120 Heidelberg, Germany

**Keywords:** head and neck squamous cell carcinoma, human papillomavirus, orthotopic tumor model

## Abstract

Head and neck squamous cell carcinomas (HNSCC) caused by infections with high-risk human papillomaviruses (HPV) are responsible for an increasing number of head and neck cancers, particularly in the oropharynx. Despite the significant biological differences between HPV-driven and HPV-negative HNSCC, treatment strategies are similar and not HPV targeted. HPV-driven HNSCC are known to be more sensitive to treatment, particularly to radiotherapy, which is at least partially due to HPV-induced immunogenicity. The development of novel therapeutic strategies that are specific for HPV-driven cancers requires tumor models that reflect as closely as possible the characteristics and complexity of human tumors and their response to treatment. Current HPV-positive cancer models lack one or more hallmarks of their human counterpart. This study presents the development of a new HPV16 oncoprotein-dependent tumor model in MHC-humanized mice, modeling the major biologic features of HPV-driven tumors and presenting HLA-A2-restricted HPV16 epitopes. Furthermore, this model was developed to be orthotopic (base of tongue). Thus, it also reflects the correct tumor microenvironment of HPV-driven HNSCC. The cancer cells are implanted in a manner that allows the exact control of the anatomical location of the developing tumor, thereby homogenizing tumor growth. In conclusion, the new model is suited to study HPV16-specific therapeutic vaccinations and other immunotherapies, as well as tumor-targeted interventions, such as surgery or radiotherapy, or a combination of all these modalities.

## 1. Introduction

Head and neck squamous cell carcinomas (HNSCC) arise from different segments of the upper aero-digestive tract. They are generally caused by exposure to tobacco and alcohol consumption or by infections with high-risk human papillomaviruses (HPV16, HPV18, etc.). In recent years, the incidence of HPV-driven (HPV^+^) HNSCC has been increasing, particularly in Western countries [1,2,3]. HPV^+^ and carcinogen-driven (HPV^−^) HNSCC are fundamentally different, both molecularly and clinically. Indeed, differences regarding mutational profile, alterations of cellular functions (metabolic, cell cycle, etc.) and treatment response have been reported. These differences have been extensively reviewed in recent years [3,4,5,6,7]. Nevertheless, current treatment strategies for both tumor entities are similar and involve surgery, radiotherapy, chemotherapy and immune checkpoint blockade depending on the stage and anatomical location of the tumor [8]. Interestingly, HPV^+^ HNSCC are more sensitive to treatment, particularly to radiotherapy. It appears that this sensitivity is not only due to the effect of HPV on cellular pathways, such as DNA repair. Indeed, HPV-associated immunogenicity of the cancer cells also significantly contributes to the sensitivity of HPV^+^ cancer cells, stressing the importance of an intact immune system [7]. Encouragingly, several ongoing clinical trials investigate treatment de-escalation strategies for HPV^+^ HNSCC. Only a few ongoing trials are investigating HPV-specific therapies, such as therapeutic vaccination. The preclinical assessment of new therapies requires not only the use of in vitro models but also in vivo models that recapitulate the complexity of the tumor microenvironment (TME) and an immune response, as well as host–tumor and host–treatment response. Indeed, the TME and the anti-tumor immune response are crucial as they determine tumor progression and treatment response [9,10,11]. Several head and neck cancer models have been developed over the years. As discussed in further detail in a review by Tinhofer and colleagues [12], in vivo models include xenografts of HPV^+^ HNSCC cells in immunocompromised mice, genetically or carcinogen-induced tumors and syngeneic cancer cell lines that can be injected in the base of the tongue. Syngeneic models, such as the 2277-NS and SCC-VII cell lines, can be genetically modified to express HPV proteins, resulting in their respective E6/7 positive counterparts PAP-A2 [13] and SCC-VII_E6/7 [14]. These models are, however, not strictly dependent on HPV as the cells were already transformed prior to the introduction of HPV oncoproteins. To the best of our knowledge, no orthotopic (base of tongue) truly HPV-dependent tumor model has been described so far in immunocompetent mice. However, it is well known that orthotopic tumor growth more faithfully reflects the characteristics of human tumors, such as invasiveness or anti-tumor immune responses [15,16,17]. Furthermore, most of the existing mouse models that express HPV proteins are suboptimal for therapeutic vaccination approaches as they express murine MHC molecules and thereby different epitopes than human cells. This limitation has already led some clinical trials to fail in reproducing promising preclinical results. For instance, most previous therapeutic vaccines were tested in the well-established TC-1 mouse model. However, this model presents a strongly immunodominant epitope, which is not the case in human HPV16-associated tumors. Therefore, vaccination approaches that succeeded in eliminating TC-1 tumors did not exhibit anti-tumor effects in human patients. Thus, to develop HPV-specific treatment options that can be tested in vivo, a new syngeneic orthotopic HPV-dependent tumor model is required in fully immunocompetent MHC-humanized mice. In this present work, an HPV16 E6/E7-dependent model was developed in A2.DR1 mice, providing a new HPV^+^ tumor model that is suited for preclinical testing of new HPV-specific treatment options, including therapeutic vaccination. 

## 2. Materials and Methods

### 2.1. Cell Lines 

E6/7-lucA2 cells are lung-cell-derived cancer cells originating from A2.DR1 mice, as previously described [17]. In brief, primary lung cells were immortalized by HPV16 E6 and E7 using lentiviral transduction. Immortalized cells were then stably transfected with an S/MAR episomal vector coding for HRAS^G12V^ and firefly luciferase. These cells were passaged in vivo and single-cell sorted, as previously described. One of the clonal cell lines was then passaged in vivo a second time to obtain the cells used for this project. The cells were cultured in RPMI supplemented with 10% FBS, 2 mM L-Glutamine, 1% penicillin/streptomycin, 2 µg/mL puromycin and 1 µg/mL blasticidin. PAP-A2 cells were previously generated in our group and were cultured, as described before [13]. TC-1/A2-luc cells (TC-1/A2 [18] in-house transduced with luciferase) were cultured in RPMI supplemented with 10% FBS, 2 mM L-Glutamine and 1% penicillin/streptomycin. HeLa-CaG-luc cells were cultured in DMEM supplemented with 10% FBS, 2 mM L-Glutamine and 1% penicillin/streptomycin. CaSki cells are cervical carcinoma cells that were cultured in DMEM supplemented with 10% FBS, 2 mM L-Glutamine and 1% penicillin/streptomycin.

### 2.2. Mice

A2.DR1 mice are transgenic for HHD (thus presenting HLA-A2:01-restricted peptides) and HLA-DR1 and are genetically modified to lack all murine MHC molecules [19,20]. The animals were provided by the Institut Pasteur (Paris, France) and were bred under specific-pathogen-free conditions. All experiments were carried out according to governmental and institutional guidelines and were authorized by the local authorities under the permit number G241-18.

### 2.3. Tumor Implantation

For tumor implantation, E6/7-lucA2 cells were injected in male and female 12–20 week old A2.DR1 mice. E6/7-lucA2 cells were cultured, as described above, and harvested during the exponential phase of proliferation. Cells were washed three times with PBS. Prior to each injection, the cell suspension was homogenized. Animals were anesthetized by isoflurane and fixed in a stereotactic frame (World Precision Instruments #502650 and #502063) using a toothbar. Mice have to be positioned in a way that the midline of the head is perpendicular to the table. Ear bars are used to ensure that the head is well fixed in this position. Cells were aspirated (World Precision Instruments #Nanofil-10) using an automated micro-injection pump (World Precision Instruments #UMP3T-1). The needle was located on the midline 9 mm caudally to the lower lip and inserted through the skin to a depth of 6 mm. In that position, 10 × 10^5^, 5 × 10^5^, 2.5 × 10^5^, 1 × 10^5^ or 0.5 × 10^5^ cancer cells were injected in 10 µL PBS using the controller of the pump at a speed of 1 µL per second. For animals that are of different size compared to 12 to 20-week-old A2.DR1 mice, the exact position of the needle might need to be adjusted. All tumor-bearing animals were sacrificed upon visible signs of distress or upon loss of > 20% body weight.

### 2.4. Western Blot

For Western blot analysis, cells were lysed using lysis buffer (10 mM tris-HCL pH 7.5, 50 mM KCl, 2 mM MgCl_2_, 1% Triton X-100, 1 mM DTT, 1 mM PMSF and protease inhibitor cocktail (Roche)). Samples were incubated for 20 min on ice with intermittent mixing and were then centrifuged for 10 min. Protein samples were mixed with 4x Laemmli-buffer-containing β-mercaptoethanol and then heated at 95 °C for 5 min. The samples were separated by SDS-PAGE and subsequently transferred to a PVDF membrane by semi-dry transfer. Membranes were blocked and then incubated overnight with primary antibodies (HPV16 E6 E6-6F4 (Euromedex), HPV16 E7 NM2 (in-house produced), Ras (G12V mutant-specific) D2H12 (Cell signaling technologies), CDKN2A/p16^INK4a^ EPR20418 (abcam) or β-actin AC-74 (Sigma-Aldrich)). HRP-coupled secondary antibodies (IgG anti-Mouse IgG (H+L)-HRPO (Dianova), IgG anti-rabbit IgG (H&L)-HRPO (Rockland Immunochemicals)) were then incubated with the membrane for 1 h. Chemiluminescence signal was detected after addition of a chromogenic substrate (VWR International # RPN2232) and detected in a Biorad Chemiluminescence Detector (Biorad #ChemiDoc). 

### 2.5. siRNA Transfection

Synthetic siRNAs (Life Technologies, Carlsbad, CA, USA) were transfected with Lipofectamine RNAiMAX (Invitrogen, Waltham, MA, USA), according to the manufacturer’s instructions, at a final siRNA concentration of 40 nM. To minimize potential off-target effects, six different siRNAs, targeting HPV16 E6 or E7, were pooled at equimolar concentrations, as detailed previously [21]. The siRNA target sequences were as follows: 16E6-1: 5´-ACCGUUGUGUGAUUUGUUA-3′; 16E6-2: 5´-GGGAUUUAUGCAUAGUAUA-3′; 16E6-3: 5´-UUAGUGAGUAUAGACAUUA-3′; 16E6/E7-1: 5′-CCGGACAGAGCCCAUUACA-3′; 16E6/E7-2: 5′-CACCUACAUUGCAUGAAUA-3′; 16E6/E7-3: 5′-CAACU-GAUCUCUACUGUUA-3′; siCrtl: 5′-CAGUCGCGUUUGCGACUGG-3′.

### 2.6. Clonogenic Assay 

For colony formation assays, cells were transfected with siRNA, as described above. Three days after the transfection, cells were reseeded and incubated for 5 days. Subsequently, cells were fixed and stained with formaldehyde and crystal violet, respectively, as previously described [22,23]. 

### 2.7. Senescence Induction Assay

E6/7-lucA2 were seeded three days after siRNA transfection and grown for 4 days. Then, cell morphology was assessed for typical characteristics of senescence (enlargement, flattening, protrusions) and senescence-associated β-galactosidase activity was detected, as previously described [22,23].

### 2.8. Flow Cytometry Assay

For the detection of HLA-A2 or HHD levels on human and murine cells, respectively, cells were harvested and labeled with an HLA-A2 FITC antibody (BD # 551285) or IgG2b, κ (isotype control) (Biolegend # 506324) for 30 min. After labeling, cells were fixed with 2.5% formaldehyde and analyzed using a FACS Canto II™ (BD). Data analysis was performed using FlowJo™ V10 software.

### 2.9. Immunoprecipitation of HLA-Displayed Peptides

HLA immunoprecipitation (IP) was performed according to previously published protocols [24,25]. Briefly, a pellet of around 1 × 10^8^ cells was lysed with a lysis buffer containing 1% N-octyl-β-D glucopyranoside, 0.25% Na-Deoxycholate, cOmplete protease inhibitor cocktail (Sigma-Aldrich, Mannheim, Germany) at 1 tablet per 5 mL and 1 mM PMSF (Carl Roth, Karlsruhe, Germany) in PBS. After centrifugation at 40,000 *g*, 4 °C, for 30 min, HLA–peptide complexes were immuno-isolated by incubation with BB7.2 mouse anti-human HLA-A2 monoclonal antibody, crosslinked to protein G Sepharose beads (Cytiva, Marlborough, MA, USA) for 4 h at 4 °C under constant mixing on a rotating wheel. Supernatant was discarded after centrifugation at 3200 *g* for 3 min at RT. Pelleted HLA–peptide complexes bound to antibody beads were washed 3 times in each of the following steps: (i) ice-cold 20 mM Tris-HCl pH 8 containing 150 mM NaCl, (ii) same buffer with 400 mM NaCl, (iii) 20 mM Tris-HCl without NaCl. Peptides were eluted from HLA bound to antibody beads by 0.3% TFA. Resulting peptides were desalted by reverse-phase purification using a SepPak 96-well plate. During desalting, an oxidation reaction was induced by 30 sec incubation with 500 µL performic acid solution (5 µL 30% H_2_O_2_ and 45 µL 10% formic acid combined for 5 min and diluted to 10 mL). The sample was eluted with 500 µL 28% ACN (acetonitrile) and dried by vacuum centrifugation.

### 2.10. LC-MS

Samples were dissolved in 4 µL 5% ACN, 0.1% TFA spiked with 50 fmol Pierce Peptide Retention Time Calibration Mixture (PRTC, Thermo Fisher Scientific, Waltham, MA, USA) by sonication for 3 min. Analysis was run by liquid chromatography (U-3000, Thermo Fisher Scientific) coupled to an Orbitrap Exploris 480 mass spectrometer (Thermo Fisher Scientific). The LC gradient consisted of a first segment increasing from 3% to 7.5% B (19.9% H_2_O, 0.1% FA, 80% ACN) and 92.5% A (0.1% FA in H_2_O) over 5 min, followed by a second segment reaching 35.6% B in 75 min. Finally, the % B was increased in 2 steps to 95% in 4 min, followed by 5 min wash before 11 min equilibration at 2% B. Protonated polycyclodimethylsiloxane (PCM-6, a background ion originating from ambient air) at 445.12 m/z served as a lock mass. For targeted epitopes, PRM scans were set to 60 k or 120 k resolution at 200 m/z, dynamic IT setting set to target 5 scans per peak and 1000% AGC target. The isolation window of ≤1 m/z and the normalized collision energy were tuned per precursor.

MS2 data were analyzed with the Skyline software (v. 21.2) [26]. Up to 10 of the most intense transitions according to a reference library gathered from stable isotope-labeled peptides were extracted with 7 ppm mass tolerance. Detected peaks were manually curated. Light peaks were discarded when peak retention times or shapes did not match with a stable-isotope labelled reference library, when the normalized spectral contrast angle (dotp) [27] was low, or when too few transitions were detected. A dotp of ≥ 0.85 at a minimum of 5 transitions was used as a cut-off for detection validation. Data visualization was performed with R (v. 4.1) [28] with the “tidyverse” suite of packages (v. 1.3.0) [29].

### 2.11. Cytotoxicity Assay 

For the VITAL-FR cytotoxicity assay [30], E6/7-lucA2 target cells and 2277-NS control target cells (that do not express the target epitope) were labeled with CSFE and Far Red fluorescence markers, respectively, and were co-cultured. Antigen-specific CD8^+^ T cells were isolated from splenocytes of A2.DR1 mice vaccinated with the E7_11–19_ peptide. These T cells were incubated with the co-culture at different effector to target (E:T) ratios. After 48 h, the cell suspension was analyzed in a BD FACS Canto II™ to determine the ratio of CFSE to Far Red-labeled cells relative to the ratio without T cells. The percentage of specific killing was calculated as follows:

$$\mathrm{Specific}\;\mathrm{killing}\;[\%]=100-\frac{\frac{\%{\mathrm{CFSE}}^{+}\;\mathrm{specific}\;\mathrm{target}\;\mathrm{cells}\;(\mathrm{with}\;T\;\mathrm{cells})}{\%\mathrm{Far}\;{\mathrm{Red}}^{+}\;\mathrm{unspecific}\;\mathrm{target}\;\mathrm{cells}\;(\mathrm{with}\;T\;\mathrm{cells})}}{\frac{\%{\mathrm{CFSE}}^{+}\;\mathrm{specific}\;\mathrm{target}\;\mathrm{cells}\;(\mathrm{without}\;T\;\mathrm{cells})}{\%\mathrm{Far}\;{\mathrm{Red}}^{+}\;\mathrm{unspecific}\;\mathrm{target}\;\mathrm{cells}\;(\mathrm{without}\;T\;\mathrm{cells})}}$$

### 2.12. Luminescence Detection

For in vitro luminescence detection, 10,000 cells were seeded in a 96-well plate and 100 µL of Luciferase Assay Substrate (Promega, Madison, WI, USA) were added to the cells. The luminescence signal was measured with a GloMax Explorer Multimode Microplate Reader.

For in vivo luminescence detection, animals were injected i.p. with 150 mg/kg D-Luciferin (Biomol, Hamburg, Germany). The radiance (photons/s) was measured at the emission maximum 12 min post-injection of the substrate using an IVIS^®^Lumina III imaging machine. The image analysis was performed using the Living Image software (Perkin Elmer, Waltham, MA, USA). 

### 2.13. Magnetic Resonance Imaging (MRI)

Nuclear MRI was carried out by the DKFZ small animal imaging core facility using a Bruker BioSpec 9.4 Tesla MRI (Ettlingen, Germany). Mice were anesthetized with 3.5% sevoflurane in air. T2 weighted imaging was performed using a T2_Turbo_RARE: TE = 33 ms, TR = 4000 ms, FOV 20 × 20 mm, slice thickness 1 mm, averages = 2, Scan Time 3 m 12 s, echo spacing 8.25 ms, rare factor 8, slices 20, image size 192 × 192. Resolution 0.104 × 0.104 mm. T1 weighted imaging was performed using a T1_RARE: TE = 6 ms, TR = 800 ms, FOV 20 × 20 mm, slice thickness 1 mm, averages = 3, Scan Time 3 m 50 s, echo spacing 6 ms, rare factor 2, slices 20, image size 192 × 192. Resolution 0.104 × 0.104 mm. Lesions were detected in T2- and T1-weighted axial and sagittal image sequences. Regions of interests (ROI) were manually selected in each slice. The tumor volumes were calculated using ImageJ (software version 1.52e).

### 2.14. Histologic Analysis

Tumors were resected and fixed in 4% paraformaldehyde prior to embedding in Paraffin. Formaline-fixed paraffine-embedded (FFPE) sections were either used for hematoxylin and eosin (HE) staining or for immunolabeling. For the latter, samples were incubated with primary antibodies CD3 (Invitrogen #MA1-90582), CD8 (Invitrogen #14-0808-82)), CD4 (eBioscience #14-9766), FoxP3 (Cell Signaling Technologies #12653), CD11b (Abcam #ab133357), Cytokeratin (Dako #Z0622), Vimentin (Abcam #ab92547), α SM actin (Sigma-Aldrich #A2547) or Desmin (Agilent #M0760). Subsequently, secondary antibody incubation was performed with HRP-coupled antibodies (Jackson Immuno Research # 312-005-045). Images were acquired using an Aperio AT2 slide scanner (Leica Biosystems) and analyzed with Aperio ImageScope v12.4.0.5043. 

## 3. Results

### 3.1. Generation and Characterization of the E6/7-lucA2 Cell Line

**Figure 1:**
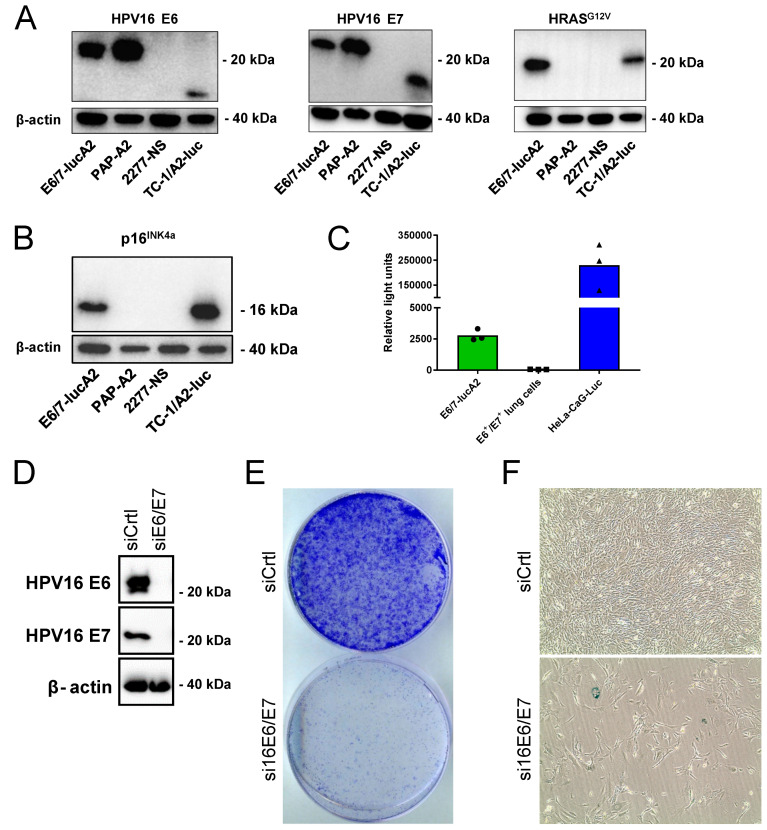
Characterization of E6/7-lucA2 cells. (**A**) Western blot analysis of HPV16 E6 and E7, as well as HRAS^G12V^ expression in E6/7-lucA2 cells (expressing tagged E6 and E7 proteins), PAP-A2 cells (A2.DR1 sarcoma cell line expressing tagged E6 and E7), 2277NS cells (HPV-negative parental cell line of PAP-A2) and TC-1/A2-luc cells (expressing non-tagged E6, E7 and HRAS^G12V^). (**B**) Western Blot analysis of p16^INK4a^ expression in indicated cell lines. (**C**) Luminescence detection after addition of D-luciferin in indicated cell lines. E6^+^/E7^+^ lung cells are the parental cell line of E6/7-lucA2. They do not express luciferase, while HeLa-CaG-luc are luciferase-expressing HeLa cells. (**D**) Western blot analysis of the expression of E6 and E7 after siRNA knock-down in E6/7-lucA2 cells. (**E**) Analysis of colony formation capacity of E6/7-lucA2 cells after transfection with non-targeting control siRNA and HP16 E6/E7-targeting siRNA pool. (**F**) Morphology analysis and detection of SA-β-Galactosidase activity in E6/7-lucA2 cells transfected with non-targeting siRNA and HP16 E6/E7-targeting siRNA.

Mirroring the generation of the widely used TC-1 mouse model of HPV16-positive cancer cells in C57BL/6 mice [31,32,33], primary lung cells were isolated as source cells from an A2.DR1 mouse. An HPV16 E6 and E7-positive cell line was generated, as previously described [17]. In brief, the primary lung cells were immortalized by transduction with the HPV16 oncoproteins E6 and E7. Subsequently, the cells were stably transfected with a plasmid vector, containing a scaffold matrix attachment region (S/MAR) for episomal replication [34,35], encoding activated HRAS and firefly luciferase. Activated HRAS is also present in TC-1 cells and is needed to render HPV-immortalized cells tumorigenic [33]. Upon two in vivo passages, the cells were expanded in vitro and characterized. Western blot analyses confirmed that the cells express the HPV16 oncoproteins E6 and E7, as well as HRAS^G12V^ (Figure 1A). Similar to naturally transformed HPV^+^ cells [36], the introduction of E6 and E7 resulted in the overexpression of p16^INK4a^ (Figure 1B). This can also be observed in E6- and E7-positive TC-1/A2-luc cells, which served as a positive control. As the sarcoma cell line PAP-A2 is not dependent on its expressed E6 and E7 oncoproteins for survival, no overexpression of p16^INK4a^ can be seen. Bioluminescence measurements validated the expression of functional luciferase, which allows in vivo tracking of tumor growth (Figure 1C). To determine if the cell line is truly HPV-dependent, siRNA knock-down of E6 and E7 was performed (Figure 1D). Upon knock-down, the cells showed a strongly reduced colony formation (Figure 1E). The down-regulation of HPV16 E6/E7 in HPV-dependent cells led to cellular senescence [22,37], which was demonstrated by a senescence induction staining. Although the assay detected only few cells that were positively stained for SA-*β*-Gal (Figure 1F), most cells presented an enlarged and flattened morphology, which is one of the characteristics of senescent cells [38]. Taken together with the results of the colony formation assay, it can be concluded that the cells entered a permanent cell cycle arrest upon the knock-down of the HPV oncogenes.

### 3.2. Analysis of HPV Epitope Presentation by E6/7-lucA2 Cells 

**Figure 2:**
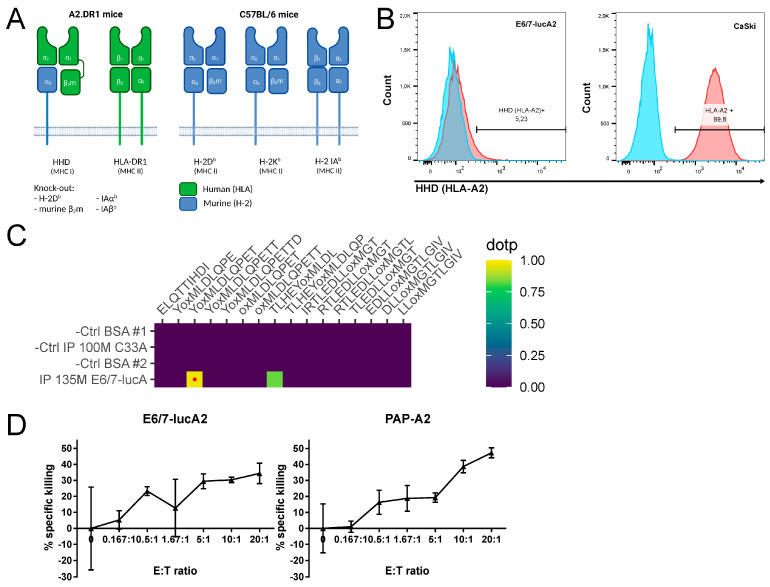
Presentation of HPV16 vaccination target epitopes by E6/7-lucA2 cells. (**A**) MHC molecules of A2.DR1 mice in comparison to C57BL/6 mice. Protein domains of human origin are marked in green and of murine origin in blue. The α3-domain of the HHD molecule is covalently linked to β2m. (**B**) Level of HHD/HLA-A2 cell surface expression on E6/7-lucA2 in comparison to CaSki cells measured by flow cytometry analysis after HLA-A2 immunolabeling. (**C**) Detection of HPV16 HLA-A2-restricted epitope YMLDLQPET (E7_11–19_) on E6/7-lucA2 HHD molecules, assessed by mass spectrometry. Left panel: Result panel for 16 targeted HLA-A2-binding peptides in HLA-A2/peptide immunoprecipitations (IP) of E6/7-lucA2 cells and HPV-negative C33A control cells. Protein matrix (BSA) control runs are performed before each sample run. NSA (normalized spectral angle) ≥ 0.85 (red dot) indicates high similarity of detected signal to synthetic reference peptide. Right panel: Extracted ion chromatogram of E6/7-lucA2 IP detection compared to the synthetic reference. (**D**) Percentage of E7_11–19_-specific killing of E6/7-lucA2 and PAP-A2 cells relative to 2277NS control cells in a VITAL-FR cytotoxicity assay. Results are presented as means of triplicate samples ± SD.

A2.DR1 mice are MHC-humanized by being knocked-out for the murine MHC class I gene H-2 D^b^ and murine β_2_m, as well as the murine MHC class II genes IAα^b^ and IAβ^b^, and by the expression of human HLA-DR1 (MHC class II) and HHD (MHC class I) (Figure 2A) [19,20]. 

HHD is a chimeric molecule comprised of α1 and α2 from HLA-A2:01 (thus presenting HLA-A2:01-restricted peptides), linked to human β_2_m and anchored in the cell membrane by murine α3, which enhances the interaction with CD8 co-receptors. Flow cytometry analysis was performed to assess the expression level of HHD on the newly developed cell line. The results show that, even though the cells express the HLA-A2 equivalent, its expression is significantly lower compared to HLA-A2 on human HPV16-transformed CaSki cells (Figure 2B). As the cells need to present HPV16 epitopes that serve as targets for therapeutic vaccination, targeted mass spectrometry analysis was used to determine if despite the low HLA-A2 expression, HPV16-derived epitopes can be detected. The results show that the HPV16 E7_11–19_ epitope can be detected on the E6/7-lucA2 cells (Figure 2C), as was previously shown for CaSki [39,40]. In order to determine if the presentation of the immunogenic E7_11–19_ epitope is sufficient for the cells to be killed by antigen-specific T cells, a VITAL-FR cytotoxicity assay was performed. Similar to the established PAP-A2 target cells (13), E6/7-lucA2 cells can be specifically killed by antigen-specific T cells (Figure 2D). Furthermore, the rate of specific killing—expressed as ratio of target to control target cells—increased with the effector to target ratio. Therefore, this new cell line is a good candidate for the preclinical testing of therapeutic vaccines.

### 3.3. Generation and Characterization of Base of Tongue Tumors Derived from E6/7-lucA2 Cells

**Figure 3:**
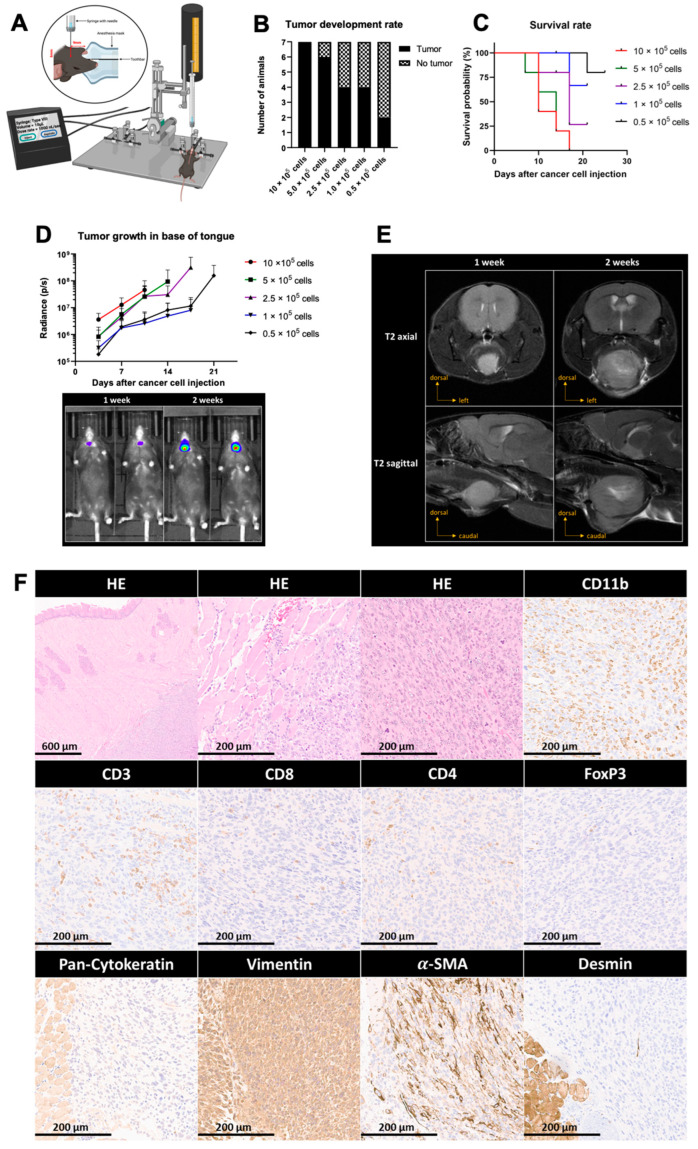
Implantation and characterization of base of tongue tumors. (**A**) Schematic representation of transcutaneous cancer cell injection into the base of the tongue using a stereotactic frame and automated microinjector pump. Mice were injected with 10 × 10^5^, 5 × 10^5^, 2.5 × 10^5^, 1 × 10^5^ or 0.5 × 10^5^ cancer cells. (**B**) Rate of tumor formation at day 14 post-injection. (**C**) Percentage of survival of animals injected with the indicated cell numbers. (**D**) Mean luminescence radiance (photons per second) of animals of each group (top) and representative IVIS images at week 1 and week 2 after cancer cell injection (bottom). (**E**) MRI analysis of base of tongue tumors shown as T2-weighed image sequences in axial and sagittal views at week 1 and week 2 after cancer cell injection. (**F**) Histologic characterization of tumor growth, invasiveness and differentiation, as well as IHC staining for cancer cell lineage markers and tumor-infiltrating immune cells. Magnification: 4× or 20×.

As discussed in the literature, orthotopic tumor models recapitulate the complex tumor environment and host–tumor response better than subcutaneous models [15,16,17]. Genetically induced head and neck tumor models and the injection of established cell lines through the oral cavity into the base of the tongue, though to a lesser extent, commonly lead to heterogeneous tumor growth and side effects. This limitation makes these models difficult for therapeutic interventions that require high accuracy, such as image-guided radiotherapy or surgical intervention. To overcome this challenge, transcutaneous stereotactic injections into the base of the tongue were performed in the present study. This technique allows the cells to be implanted in the same precise anatomical location in all mice (Figure 3A). To achieve reproducible tumor implantation and growth, the correct positioning of the animals must be ensured. In addition, the cell suspension has to be homogenized before filling the syringe for each animal as high densities of cells and fast sedimentation can otherwise alter the number of injected cells. A titration of the cells was performed to determine the number of cells necessary for a high tumor formation rate, as well as a growth speed that allows therapeutic intervention before the wellbeing of the animals is compromised or they reach a humane endpoint. Using the technique mentioned above, 10 × 10^5^, 5 × 10^5^, 2.5 × 10^5^, 1 × 10^5^ or 0.5 × 10^5^ cancer cells were injected in the base of the tongue. Tumor development was assessed by bioluminescence imaging. The frequency of tumor growth increased with the number of cells that were injected (Figure 3B). We found that the group receiving 10 × 10^5^ cells was the only one in which all animals formed tumors, indicating that this amount of cells is preferable to achieve a maximal tumor growth rate. As expected, tumor-bearing mice having received higher cell numbers reached a humane endpoint quicker than mice having received lower cells numbers (Figure 3C). Thus, the lower numbers of cells might be preferable if experiments require a longer life span. The growth rate, determined by bioluminescence imaging, was not directly proportional to the number of injected cells (Figure 3D). Tumor-bearing animals were examined by nuclear magnetic resonance imaging (MRI). The results show that the tumor was located in the base of the tongue and that the tumor can be clearly distinguished from the surrounding tissue. All tumor-bearing animals were sacrificed upon reaching an endpoint criterion. At some point, all tumor-bearing animals developed a very rapid weight loss (>20%) at which point they were sacrificed according to the animal experimentation protocol. Prior to this weight loss, none of the animals presented signs of distress or unusual behavior. Histologic analysis was performed to characterize these base of tongue tumors (Figure 3F). Hematoxylin and eosin (HE) stainings of the base of the tongue showed predominantly well-circumscribed and mass-forming tumor nodules surrounded by skeletal muscle tissue. Tumor nodules were composed of fascicles of eosinophilic spindled cells with pleomorphic, blunt-ended nuclei. Additionally, several mitotic figures could be observed per high-power field (HPF). In subsequent immunohistochemical stainings, the tumor cells exhibited a protein expression profile compatible with a smooth muscle cell-like differentiation with a diffuse and strong immunoreactivity for vimentin, a patchy but strong immunoreactivity for alpha-smooth muscle actin (a-SMA) and a negative immunoreactivity for desmin, as well as pan-cytokeratin. This suggests that the E6/7-lucA2 cell line does not have an epithelial but a smooth muscle origin. Immunohistochemical characterization of the immune infiltrate showed a mixed infiltrate composed of myeloid cells (CD11b) and T lymphocytes (CD3). Among the latter, both CD8+ and CD4+ T cells were found with only sparse regulatory T cells (FoxP3).

## 4. Conclusions

HPV-driven head and neck cancers, particularly oropharyngeal squamous cell carcinomas, are rising in incidence and are often detected in late stages when limited treatment options are available. For the development of new therapeutic strategies specific for HPV-driven HNSCC, models are required that are able to reflect important features of human tumors as closely as possible. Such characteristics include the complexity of the tumor microenvironment, as well as the host–tumor and host–treatment response. In this context, this study established and characterized a new orthotopic base of tongue tumor model. Since it was shown that this model is not of epithelial origin, it does not necessarily reflect the cell biological characteristics of squamous cell carcinomas. However, this model is well suited for the preclinical assessment of new immunotherapeutic approaches for HPV-driven cancer as it fulfills several important criteria: First, the E6/7-lucA2 cancer cell line was immortalized by HPV16 E6 and E7 oncoproteins and truly depends on their expression, as is the case for naturally HPV-transformed cancer cells. Similar to human HPV-transformed cells and the murine TC-1/A2-luc tumor model, p16^INK4a^ is overexpressed, again indicating the functionality of the oncogenes. Our data also indicate that cancer cells cannot silence or mutate the HPV transgenes as this would result in cell death. Second, using stereotactic injection, tumor growth can be restrained to a specific anatomical location, here at the base of the tongue. Thereby, it overcomes the heterogeneous tumor growth of some other models, such as genetically or carcinogen-induced head and neck cancers. As E6/7-lucA2 cells express firefly luciferase, tumor growth can be monitored on a regular basis. This tracking of the tumor growth can be used to assess the treatment efficacy during preclinical studies. The MRI analysis of the tumor allows the precise localization of the tumors and the separation of tumors and healthy tissue. This precision is important for some therapeutic interventions, such as image-guided stereotactic radiotherapy or surgery, which are commonly combined with immunotherapy approaches. Third, the E6/7-lucA2 cell line was derived from primary cells isolated from MHC-humanized A2.DR1 mice. Although the expression of HHD (HLA-A2) is much lower than the expression of HLA-A2 in established HPV-transformed human cell lines, immunogenic HLA-A2-restricted HPV epitopes can be detected on the surface of the cells. Thus, this model can be used for the preclinical testing of therapeutic HPV vaccines using epitopes that are restricted to HLA-A2, the most common HLA supertype [41]. The time span for therapeutic interventions provided by this model is regarded as sufficient as the animals did not show any signs of distress or impairment prior to the sudden weight loss, at which point they needed to be sacrificed. Furthermore, in an implantable animal model it is also possible to administer some immunotherapy approaches (especially vaccines) before tumor cell implantation to allow the assessment of the potency of tumor-specific memory responses. Altogether, this study provides a new HPV-transformed mouse model of base of tongue tumors that shares important characteristics with human HPV-driven head and neck tumors and that is suited for the preclinical assessment of novel HPV-targeted immunotherapeutic strategies. 

## Data Availability

The data presented in this study are contained in this article. MS raw data are available on request from the corresponding author.
